# Cost-of-illness analysis reveals potential healthcare savings with reductions in type 2 diabetes and cardiovascular disease following recommended intakes of dietary fiber in Canada

**DOI:** 10.3389/fphar.2015.00167

**Published:** 2015-08-11

**Authors:** Mohammad M. H. Abdullah, Collin L. Gyles, Christopher P. F. Marinangeli, Jared G. Carlberg, Peter J. H. Jones

**Affiliations:** ^1^Department of Human Nutritional Sciences and Richardson Centre for Functional Foods and Nutraceuticals, University of Manitoba, Winnipeg, MBCanada; ^2^Department of Agribusiness and Agricultural Economics, University of Manitoba, Winnipeg, MBCanada; ^3^Pulse Canada, Winnipeg, MBCanada

**Keywords:** fiber consumption, type 2 diabetes, cardiovascular disease, economic benefits, healthcare cost saving, nutrition economics, public health

## Abstract

**Background:** Type 2 diabetes (T2D) and cardiovascular disease (CVD) are leading causes of mortality and two of the most costly diet-related ailments worldwide. Consumption of fiber-rich diets has been repeatedly associated with favorable impacts on these co-epidemics, however, the healthcare cost-related economic value of altered dietary fiber intakes remains poorly understood. In this study, we estimated the annual cost savings accruing to the Canadian healthcare system in association with reductions in T2D and CVD rates, separately, following increased intakes of dietary fiber by adults.

**Methods:** A three-step cost-of-illness analysis was conducted to identify the percentage of individuals expected to consume fiber-rich diets in Canada, estimate increased fiber intakes in relation to T2D and CVD reduction rates, and independently assess the potential annual savings in healthcare costs associated with the reductions in rates of these two epidemics. The economic model employed a sensitivity analysis of four scenarios (universal, optimistic, pessimistic, and very pessimistic) to cover a range of assumptions within each step.

**Results:** Non-trivial healthcare and related savings of CAD$35.9-$718.8 million in T2D costs and CAD$64.8 million–$1.3 billion in CVD costs were calculated under a scenario where cereal fiber was used to increase current intakes of dietary fiber to the recommended levels of 38 g per day for men and 25 g per day for women. Each 1 g per day increase in fiber consumption resulted in annual CAD$2.6 to $51.1 million savings for T2D and $4.6 to $92.1 million savings for CVD.

**Conclusion:** Findings of this analysis shed light on the economic value of optimal dietary fiber intakes. Strategies to increase consumers’ general knowledge of the recommended intakes of dietary fiber, as part of healthy diet, and to facilitate stakeholder synergy are warranted to enable better management of healthcare and related costs associated with T2D and CVD in Canada.

## Introduction

Globally, the growing prevalence of type 2 diabetes (T2D) and CVD has been accompanied by escalating costs related to healthcare and society’s loss of productivity, putting these two diet-related epidemics among the world’s top public health policy priorities. Approximately 9% of adults around the world were diagnosed with diabetes in World Health Organization (2014), of which 90% had T2D, together with 4.9 million deaths and diabetes-related costs that reached US$612 billion. In Canada, the prevalence of diagnosed diabetes increased by 70% between 1998/99 and 2008/09 (Public Health Agency of Canada, 2011), with a conservative total cost estimate of CAD$2.5 billion (excluding costs of complications) in 2000 and total direct healthcare costs projected to increase to over CAD$8 billion annually by 2016 (Ohinmaa et al., 2004). Similar trends have been observed for costs related to CVD, which accounted for over 30% of all deaths (17 million) worldwide in 2008 (World Health Organization, 2011). The global economic burden associated with the disease management costs of CVD was estimated to reach US$863 billion in 2010 and is projected to exceed 1 trillion by 2030 (World Health Organization, 2011). In Canada, 29% of all deaths in 2008 were secondary to CVD, which was estimated to cost upward of CAD$21 billion in annual healthcare expenditures (Conference Board of Canada, 2010).

Dietary behaviors consistent with guidelines for healthy eating have the potential to produce substantial health and economic benefits. Previous research has demonstrated that, for example, increased consumption of dairy, as well as reduced intakes of calories, sodium, and saturated fat would facilitate considerable health and economic benefits ranging from US$2 to $58 billion annually (McCarron and Heaney, 2004; Dall et al., 2009; Bibbins-Domingo et al., 2010). From a functional food perspective, Gyles et al. (2010) showed that direct and indirect coronary heart disease (CHD)-related costs could be reduced by CAD$38 million to $2.5 billion if Canadians increased intakes of phystosterols. More recently, Schmier et al. (2014, 2015) modeled the potential for substantial constipation-related savings in healthcare costs in the US and Europe from increased consumption of dietary fiber.

In Canada, the definition of dietary fiber includes carbohydrates that naturally occur in foods of plant origin and are not digested or absorbed by the small intestine of humans (Health Canada, 2012a). Among other disorders, dietary fiber has been associated with meaningfully lower prevalence of T2D and CVD (Merchant et al., 2003; Kendall et al., 2010; Chen et al., 2013). For instance, a meta-analysis of five cohorts (*n* = 239,485) showed a 19% lower risk of diabetes (RR = 0.81, 95% CI 0.70–0.93) among individuals in the highest quintile of dietary fiber intake (Anderson and Conley, 2007). Similarly, analysis of seven cohorts (*n* = 158,327) demonstrated that, compared to the lowest quintile of fiber intake, the highest levels of dietary fiber reduced risk of CHD by 29% (RR = 0.71, 95% CI 0.47–0.95; Anderson, 2004). Further systematic analysis of the available evidence has suggested that, compared to fruit or vegetable-derived fiber, diets with higher levels of fiber from cereals are associated with the greatest reduction in risk for T2D (Cho et al., 2013; InterAct Consortium, 2015) and CVD (Mozaffarian et al., 2003; Threapleton et al., 2013).

The average level of fiber consumed by Canadians is estimated to be 19.1 and 15.6 g per day for males and females, respectively (Belanger et al., 2014), and are well-below the IOM recommended adequate intakes for males (38 g per day) and females (25 g per day) between 19 and 50 years of age (Institute of Medicine, 2002). Sources of dietary fiber within the Canadian food supply are plentiful as both whole foods and fiber-fortified foods. Additionally, the Canadian population already has the necessary tools available in the marketplace to enact behavioral changes that would be consistent with increased intakes of dietary fiber. However, fiber education and motivation remain as long-term dietary challenges to consciously increase the consumption of fiber in Canada.

Given Canada’s publically funded healthcare system, the promotion of dietary strategies that facilitate meaningful reductions in healthcare costs and prolong economic productivity can be considered a powerful tool for healthcare practitioners and policymakers attempting to manage economic resources. In this regard, the potential economic impact of increasing Canadians’ fiber consumption can be calculated by determining the proportion of the economic burden related to T2D and CVD that can be avoided by increasing the consumption of dietary cereal fiber. Thus, the objective of this study was to evaluate the potential economic benefits of increased intakes of dietary cereal fiber for adults as determined by consequence reductions in annual healthcare costs associated with independently reduced rates of T2D and CVD in Canada.

## Materials and Methods

### Study Design

Utilizing data from the current medical literature and recent healthcare cost estimates from national databases, a three-step variation of a cost-of-illness analysis was conducted to evaluate the healthcare-related economic benefits of fiber consumption: (i) Determination of the *success rate*, which represents the proportion of the Canadian population expected to consume fiber-rich diets, (ii) Independent analysis of the *T2D and CVD reduction rate*s that would result from consumption of dietary fiber, and (iii) Estimation of the *healthcare cost savings* associated with reductions in T2D and CVD rates. Additionally, a sensitivity analysis of four scenarios (universal, optimistic, pessimistic, and very pessimistic) was created to cover a range of predictions within each of these steps.

Overall, three different sets of analyses were completed. The first analysis reflected the cost reductions in T2D and CVD-related healthcare services when cereal fiber is utilized to increase current actual intakes of dietary fiber for Canadian men (19.1 g per day) and women (15.6 g per day; Belanger et al., 2014) to the IOM’s adequate intakes of 38 g per day and 25 g per day for men and women, respectively (Institute of Medicine, 2002). These are the cut-off values that policy makers, dietitians, and other healthcare providers in Canada and the US typically use as guidelines. The second analysis examined the healthcare cost savings per g increase in cereal fiber intake. The third analysis estimated the total dollar savings at incremental levels of 20, 25, 30, and 35 g fiber per day for men and women alike, reflecting a moderate stepwise increase in cereal fiber consumption. **Table 1** summarizes the input parameters of the model. This analysis was applied to Canadian adults, which were defined as men and women ≥18 years of age. Demographic populace data was from 2014 and attained from Statistics Canada (Statistics Canada, 2015).

**Table 1:** Summary of the input parameters for the cost saving assessment model.

Parameter	Men	Women	Source
Current fiber intake, g per day	19.1	15.6	Belanger et al. (2014)
Target fiber intake, g per day			
	38	25	Institute of Medicine (2002)
	20 25 30 35	20 25 30 35	Assumption for the economic valuation of incremental increases in intake
	20.1	16.6	Assumption for economic valuation of a per g increase in intake
T2D reduction per 1 g cereal fiber intake, %	2.5	2.5	InterAct Consortium (2015)
CVD reduction per 1 g cereal fiber intake, %	1.1	1.1	Threapleton et al. (2013)

*CVD, cardiovascular disease; IOM, Institute of Medicine; T2D, type 2 diabetes.*

### Step 1 of the Cost-of-Illness Analysis: *Assessing the Success Rate*

In economic theory, individuals make choices to maximize their utility, an unobservable metric for the satisfaction they receive from consuming goods or receiving services, subject to the constraints of time and financial resources. Previous research has explored how consumers maximize their utility in the context of food, nutrition, and health [Grossman (1972, March–April); Blaylock et al., 1999; Cawley, 2004]. More recent work (Lioutas, 2014) has examined the information processing behavior that consumers utilize as it relates to food choices. When an individual processes available dietary information and, based on this information, chooses to purchase and consume “healthy” foods, the individual is maximizing their utility. If this behavior is sustained over a period of time, the consumer may experience a health improvement.

The eventual economic benefit of increased dietary fiber intake is dependent on individual consumer decisions within the marketplace. Therefore, any model which attempts to measure the potential public health benefits should start with an examination of consumer behavior. A key assumption in health psychology and economics is that behavioral changes are the result of a decision-making process, where the benefits and costs of particular changes are considered before the adoption of a specific course of action. In previous short-term research that assessed consumer behavior in relation to dietary fiber intakes and health claims, fiber was found to be viewed favorably (Mialon et al., 2002; Dean et al., 2007; Baixauli et al., 2009; Ginon et al., 2009; Tudoran et al., 2009). For instance, when grain products were labeled as high in fiber and provided to consumers, enhanced likelihood of consumption (Mialon et al., 2002) as well as higher acceptability and purchase intentions (Baixauli et al., 2009) were reported. However, the actual long-term intake behavior for dietary fiber has not been previously measured. As such, in order to estimate the percent of the population expected to adopt a fiber-rich diet in Canada, a sensitivity analysis of universal, optimistic, pessimistic, and very pessimistic success rate scenarios was modeled based on findings from previous research (Mialon et al., 2002; Dean et al., 2007; Baixauli et al., 2009; Ginon et al., 2009; Tudoran et al., 2009). The universal fortification scenario assumed a 100% success rate and represented a dramatic shift in the dietary habits of Canadians. While this scenario is not realistically achievable in the short-term, it represents the maximum potential of economic savings with increased fiber intake over the very long-term. The optimistic success rate was assumed to be 50%, and represented a medium- to short-term pragmatic estimate of the potential economic savings possible through an increased dietary fiber intake. The pessimistic and very pessimistic success rates were set at 15% and 5% to respectively represent a less positive (but practical) short-to-medium term estimate of economic savings following increased fiber intake, and determine the impact on the cost estimates when assumptions are more pessimistic than normal.

### Step 2 of the Cost-of-Illness Analysis: *Estimating Disease Reduction due to Increased Dietary Cereal Fiber Intake*

Several epidemiological and dietary intervention studies of different designs have documented beneficial impacts of increased dietary fiber intake on disease risk and mortality rates. Similar to Step 1 of this analysis, we established possible scenarios regarding reductions in the incidence of T2D and CVD with higher intakes of dietary fiber based on the current English-language medical and nutritional literature. After careful examination of the available literature, model assumptions were generated based on two recent comprehensive systematic meta-analyses by Threapleton et al. (2013) and InterAct Consortium (2015). Eligibility criteria and quality of studies are included in the meta-analyses of choice. Under the context of this study and the studies reviewed, cereal fiber refers to fiber from cereal grains.

#### Estimated Effect of Increased Dietary Cereal Fiber on Prevalence of Type 2 Diabetes

The inverse relationship between increased dietary fiber and rates of T2D risk were estimated from a meta-analysis by InterAct Consortium (2015) based on data from prospective studies, where a per 10 g increase in cereal fiber resulted in an average of 25% reduced risk of T2D (RR = 0.75, 95% CI 0.65–0.86). These results are corroborated by a previous systematic review by Cho et al. (2013), where cereal fiber consumption was estimated to decrease the risk of T2D by 18–40%.

#### Estimated Effect of Increased Dietary Cereal Fiber on Prevalence of Cardiovascular Disease

Similar to the T2D component, data for the estimated CVD risk reduction with higher intakes of dietary fiber was derived from a systematic review and meta-analysis of cohorts by Threapleton et al. (2013). There, based on a pooled estimate from prospective studies, consumption of cereal fiber intake was found to be inversely associated with risk of CVD (RR = 0.92 per 7 g per day, 95% CI 0.84–1.0), equivalent to a 1.1% lower CVD risk per g cereal fiber consumed.

Based on the meta-analytic data from Threapleton et al. (2013) and InterAct Consortium (2015), our analysis assumed that for each 1 g increase in dietary cereal fiber, incidence of T2D and CVD would be conservatively decreased by 2.5 and 1.1%, respectively (**Table 1**). For the purpose of this modeling exercise, it is assumed that the relative risk reduction of T2D and CVD per g cereal fiber intake corresponds to a decrease in the population-wide incidence of T2D and CVD of the same magnitude.

### Step 3 of the Cost-of-Illness Analysis: *Estimating the Potential Annual Savings in Costs Associated with Type 2 Diabetes and Cardiovascular Disease*

Individual consumers are the primary beneficiary of any health improvement following increased consumption of dietary fiber. However, in a publicly funded healthcare system, such as the system adopted by Canada, more widespread benefits can also be expected. A reduction in T2D and CVD rates will result in the diversion of fewer resources to the treatment of these highly prevalent health conditions. Given a subsequent reduction in morbidity and mortality that are secondary to improved dietary habits of the population, society will also benefit from increased productivity. In this way, although consumers make deliberate dietary choices, such as increasing fiber intake, without considering the effect on healthcare expenses, the overall impact of their actions on society can be considerable.

The economic cost of disease in Canada is generally broken down into direct and indirect categories. Direct costs are those incurred by the healthcare system with the goal of improving and/or preventing a patient’s health status from deteriorating. These usually include hospital care, drug, physician visits, and, sometimes, other “miscellaneous” costs. Indirect costs, on the other hand, are commonly known as those incurred by the loss of productivity to society arising from mortality and morbidity. A detailed description of the calculations used to determine the direct and indirect disease costs in Canada can be found in the *EBIC 2005–2008* Report (Public Health Agency of Canada, 2014).

#### Overview of Type 2 Diabetes Costs

The *EBIC 2005–2008* report (Public Health Agency of Canada, 2014) recently provided a comprehensive overview of the cost of diabetes in Canada. The Statistics Canada Consumer Price Index (health and personal care sub-index) was used to inflate the 2008 estimate of CAD$2.3 billion to 2014 levels and yielded CAD$2.5 billion as the best estimated total direct and indirect economic costs of T2D (**Table 2**).

**Table 2:** Estimated direct and indirect costs of type 2 diabetes in Canada (CAD $).

Cost category	2008* ($millions)	2014^†^ ($millions)
**Direct costs**		
Hospital	492.7	527.2
Physician	487.3	521.4
Drugs	1,198.2	1,282.1
**Total direct costs**	**2,178.2**	**2,330.7**
**Indirect costs**		
Mortality	12.3	13.2
Morbidity	132.9	142.2
**Total indirect costs**	**145.2**	**155.4**

**Total costs**	**2,323.4**	**2,486.0**

*From the *EBIC 2005–2008* report (Public Health Agency of Canada, 2014).

*^†^Current dollars based on adjustments of inflation rates according to Statistics Canada Consumer Price Index.*

Estimated at CAD$1.3 billion in 2014 dollars, drugs constituted the largest direct costs of T2D, which includes the costs of prescribed and non-prescribed medications purchased in retail stores. Since many different medications are used to treat T2D, it is reasonable that a decrease in the overall incidence of the disease would subsequently lead to a decrease in T2D drug-related costs. Hospitalization costs of T2D, estimated at CAD$527.2 million in 2014 dollars, were calculated on the basis of bed occupation and aggregated by diagnostic category. Generally, hospital costs are largely the fixed costs related to operating and maintaining hospital facilities, as well as the salaries of the medical professionals and support staff. The more variable components of hospitalization include the cost of medications administered to the hospitalized patients, the cost of food and accommodation, and the cost of diagnostic procedures carried out in hospitals. A reduction in the incidence of T2D would be anticipated to lead to fewer hospitalizations resulting from this disorder and, as a consequence, reductions in variable costs. Finally, the physician costs were calculated based on fee-for-service billings submitted to provincial health insurance plans and are allocated on the basis of the primary diagnostic category. For example, if a patient visits a physician’s office for a follow-up treatment after a first diagnosis of T2D, this cost would be attributed to the cost of T2D. The estimated cost of T2D-related visits to physicians was approximately CAD$521.4 million in 2014 dollars. It follows that a reduction in overall T2D levels will result in fewer doctor visits, which will reduce these costs. Total indirect costs for T2D were estimated at CAD$155.4 million in 2014 dollars, including $13.2 million for mortality and $142.2 million for morbidity. In estimating the mortality costs of T2D, *EBIC 2005–2008* report utilized the friction cost approach, which assumes that sick and deceased workers can be replaced after a certain period of time known as the ‘friction period.’ Cost estimates of this method are normally lower than those derived from the classical human capital method. A reduction in the incidence of T2D is assumed to lead to decreases in both components of the indirect costs.

#### Analysis of Type 2 Diabetes Cost Reduction

The reduction in the cost of T2D was assumed to be linear when a decline in T2D incidence is observed. The exception was for the variable costs related to hospitalization. As described earlier, fixed and variable costs exist in hospitalization. The former is incurred regardless of the prevalence of any disease, whereas the latter is largely affected by the number of admissions. A comprehensive breakdown of hospital care costs in Canada is, to our knowledge, not available. As a result, it was necessary to approximate the portion of hospital costs that are fixed and not affected from reduced incidence of T2D. Previous research has provided a breakdown of fixed and variable costs in American hospitals and found that hospital costs are approximately 84% fixed and 16% variable (Roberts et al., 1999). Thus, for the purpose of this research, it was assumed that a reduction in T2D would not result in reductions in fixed costs of hospitalization, but would facilitate a proportional reduction in the variable costs. This means that each 1% reduction in the incidence of T2D would be followed by a 0.16% reduction in hospital costs.

It is reasonable to assume that fewer individuals with T2D will require less medication for treatment. As such, a proportional reduction was assumed for drug costs. Similarly, given that the physician care costs are based on physician billings, which are in turn based on T2D patient visits to doctors’ offices, a reduction in T2D was assumed to lead to a proportional reduction in T2D-related physician costs. Finally, as the number of cases with T2D decreases, costs associated with mortality and morbidity were assumed to follow in a proportional manner. A summary of the relationship between T2D incidence and associated costs is provided in **Table 3**.

**Table 3:** Summary of direct and indirect cost reductions that correspond to a 1% decrease in the incidence of type 2 diabetes.

Cost category	% Reduction
**Direct costs**	
Hospital*	0.16%
Drugs	1.00%
Physician care	1.00%
**Indirect costs**	
Mortality	1.00%
Morbidity	1.00%

*Based on the estimation that 16% of hospitalization costs are variable (i.e., medications and supplies) and 84% are fixed (i.e., salaries, buildings, and equipments; Roberts et al., 1999).

#### Overview of Cardiovascular Disease Costs

The *EBIC 2005–2008* (Public Health Agency of Canada, 2014) and the *NHEX 1975–2013* (Canadian Institute for Health Information, 2013) expense figures were both used as the foundation of the estimates of CVD in this analysis; again with adjustments to 2014 dollars using the Statistics Canada Consumer Price Index (health and personal care sub-index). Inflating values from the most recent 2008 estimate of CAD$12.2 billion to 2014 dollars yielded a revised valuation of CAD$13.0 billion as the best estimated economic cost of CVD in Canada. In the case of CVD, the direct costs as presented by the *EBIC 2005–2008* report include hospital care, drug, and physician visits, whereas the indirect costs include mortality and morbidity. These costs are explained further in the subsections below and summarized in **Table 4**.

**Table 4:** Estimated direct and indirect costs of cardiovascular disease in Canada (CAD $).

Cost category	2008* ($millions)	2014^†^ ($millions)
**Direct costs**		
Hospital	5,068.0	5,422.8
Drugs	4,272.7	4,571.8
Physician care	2,352.0	2,516.6
Other direct‡	134.1	143.5
**Total direct costs**	**11,826.8**	**12,654.7**
**Indirect costs**		
Mortality	92.4	98.9
Morbidity	269.6	288.5
**Total indirect costs**	**362.0**	**387.3**

**Total costs**	**12,188.8**	**13,042.0**

*From the *EBIC 2005–2008* report (Public Health Agency of Canada, 2014).

*^†^Current dollars based on adjustments of inflation rates according to Statistics Canada Consumer Price Index. ‡Represents cost estimates for Other Professionals (chiropractors, physiotherapists, private duty nurses, etc.) and Other Health Spending (home care, medical transportation, etc.) by the *National Health Expenditure Trends 1975–2013* report (Canadian Institute for Health Information, 2013) where total Other Direct costs for all diseases in 2008 reached CAD$53.0 billion. Percentage of CVD relative to total Other Direct costs within the *EBIC 1998* report (Public Health Agency of Canada, 1998) of 0.3% was used to estimate the 2008’s CVD-related other direct economic valuation of CAD$134.1 million and then adjust it to a 2014 estimate of CAD$143.5 million.*

The largest direct costs associated with CVD were the hospitalization costs, which were estimated at slightly more than CAD$5.4 billion in 2014 dollars. Again, the fixed costs associate with operating hospital facilities and the staff salaries, while the variable costs associate with drugs administered to the hospitalized patients, food, and diagnostic procedures. Similar to T2D estimates, only the variable costs are expected to decrease with fewer hospitalizations resulting from reductions in the incidence of CVD. Prescribed drug costs of CVD were estimated at CAD$4.6 billion in 2014 dollars and, similar to the T2D estimates, are logically expected to decrease with better management of the disease. The physician care costs were CAD$2.5 billion in 2014 dollars and, similar to the drug costs, are expected to decrease with fewer physician visits when CVD rates decrease. Finally, the “other direct costs” associated with CVD were estimated at CAD$143.5 million in 2014 dollars and are based on estimates of services of other health professionals (e.g., physiotherapists), public health, administration, and ambulance services from the *NHEX 1975–2013* report (Canadian Institute for Health Information, 2013). A reduction in CVD will reduce the demand for some, but not all, of the above-mentioned services and, as a result, reduces a portion of this component of direct costs.

In estimating the CVD-related mortality costs of CAD$98.9 million in 2014 dollars, similar to the analysis for T2D by *EBIC 2005–2008*, these costs were derived using the friction cost approach. It is logical that a reduction in the incidence of CVD and the corresponding decrease in mortality will reduce this cost. Morbidity, or disability, costs arise when productivity is lost due to illness for a period of time. The estimated economic burden of morbidity resulting from CVD in the *EBIC 2005–2008* report was CAD$288.5 million in 2014 dollars. Akin to the mortality component, morbidly costs are expected to decrease with a reduction in the incidence of CVD.

#### Analysis of Cardiovascular Disease Cost Reduction

Similar to the T2D cost reduction, with lower CVD rates, our CVD cost reduction analysis assumed a proportional reduction in the variable (but not the fixed) hospitalization costs, drug costs, and physician costs (**Table 5**). Unlike the previous report in Public Health Agency of Canada (1998), the new *EBIC 2005–2008* report does not provide cost estimates for services by “other health providers” for CVD, which necessitated estimations using figures from the *NHEX 1973–2013* and *EBIC 1998* reports as previously described. The costs for “Other” (5.4%), within the “Other Professionals” category (Canadian Institute for Health Information, 2013), which includes chiropractors, physiotherapists, private duty nurses, and others, were expected to be reduced in a manner proportional to the overall CVD reduction. Similarly, costs that will likely be reduced by a reduction in CVD include home care, medical transportation, training of health workers, and others (13.8%) within the *NHEX* “Other Health Spending” category. The final result was that a 1% decrease in the incidence of CVD will result in a 0.19% reduction in other direct CVD-related costs.

**Table 5:** Summary of direct and indirect cost reductions that correspond to a 1% decrease in the incidence of cardiovascular disease.

Cost category	% Reduction
**Direct costs**	
Hospital*	0.16%
Drugs	1.00%
Physician care	1.00%
Other direct^†^	0.19%
**Indirect costs**	
Mortality	1.00%
Morbidity	1.00%

*Based on the estimation that 16% of hospitalization costs are variable (i.e., medications and supplies) and 84% are fixed (i.e., salaries, buildings, and equipments; Roberts et al., 1999). ^†^Based on total disease cost by the *EBIC 2005–2008* report (Public Health Agency of Canada, 2014) and estimates from the *National Health Expenditure Trends 1975–2013* report (Canadian Institute for Health Information, 2013) for Other Professionals (5.4%) and Other Healthcare Spending (13.8%) categories.

Finally, also similar to the T2D case, morbidity and mortality costs were assumed to have a directly proportional CVD-reduction to cost-reduction relationship. Basically, since the incidence of CVD will decrease, it is a reasonable assumption that a proportional reduction in the deaths and disability from CVD will be observed. As a consequence, the loss of human capital that would ordinarily be incurred from CVD-related death and disability does occur and this facilitates an economic benefit.

## Results

**Tables 6** and **7** summarize the potential T2D and CVD direct and indirect cost savings when current levels of fiber intake (19.1 g per day for men and 15.6 g per day for women; Belanger et al., 2014) are increased with cereal fiber to levels that correspond to the IOM’s recommended adequate intakes. Under the universal fortification scenario, assuming a 100% success rate and maximum potential of economic savings over the very long run, our analysis predicted total annual healthcare and related savings of CAD$718.8 million for T2D and $1.3 billion for CVD costs. The optimistic scenario, which assumed a 50% success rate and medium- to short-term savings, on the other hand, predicted savings of CAD$359.4 million for T2D and $647.8 million for CVD costs annually. With a 15% success rate and a less positive, yet still practical, short- to medium-term effects, the pessimistic scenario showed savings of CAD$107.8 million for T2D and $194.4 million for CVD costs. Finally, the very pessimistic scenario that assumed a worst-case estimate of a 5% success rate suggested total annual savings of CAD$35.9 million for T2D and $64.8 million for CVD costs.

**Table 6:** Potential savings in type 2 diabetes costs among Canadian adults from improved intake of dietary cereal fiber (CAD $million).

	Scenario			
Cost category	Universal	Optimistic	Pessimistic	Very pessimistic
**Direct cost savings**				
Hospital	29.7	14.8	4.5	1.5
Physician	183.4	91.7	27.5	9.2
Drugs	451.0	225.5	67.7	22.6
**Total direct cost savings**	**664.1**	**332.1**	**99.6**	**33.2**
**Indirect cost savings**				
Mortality	4.6	2.3	0.7	0.2
Morbidity	50.0	25.0	7.5	2.5
**Total indirect cost savings**	**54.7**	**27.3**	**8.2**	**2.7**

**Total cost savings**	**718.8**	**359.4**	**107.8**	**35.9**

*Data represent T2D-related economic savings from the utilization of cereal fiber for increasing dietary fiber consumption from current levels (**Table 1**) to levels that correspond to the IOM’s adequate intake cut-offs, estimated at 38 g per day for men and 25 g per day for women (Institute of Medicine, 2002). The universal fortification represents the best-case scenario of potential cost savings if all Canadian adults (≥18 years of age) were to consume the adequate quantities of dietary fiber. The optimistic scenario is a medium- to short-term pragmatic estimate of the potential cost savings when 50% of adults increase intakes of dietary fiber. The pessimistic scenario is a practical short- to medium-term estimate of cost savings that could follow the increase in dietary cereal fiber intakes among 15% of adults. The very pessimistic scenario represents the worst-case estimate when up to 5% of adults make the dietary change.*

**Table 7:** Potential savings in cardiovascular disease costs among Canadian adults from improved intake of dietary cereal fiber (CAD $million).

	Scenario			
Cost Category	Universal	Optimistic	Pessimistic	Very pessimistic
**Direct cost savings**				
Hospital	134.3	67.2	20.1	6.7
Drugs	707.7	353.8	106.1	35.4
Physician care	389.5	194.8	58.4	19.5
Other direct	4.2	2.1	0.6	0.2
**Total direct cost savings**	**1,235.7**	**617.9**	**185.4**	**61.8**
**Indirect cost savings**				
Mortality	15.3	7.7	2.3	0.8
Morbidity	44.7	22.3	6.7	2.2
**Total indirect cost savings**	**60.0**	**30.0**	**9.0**	**3.0**

**Total cost savings**	**1,295.7**	**647.8**	**194.4**	**64.8**

*Data represent CVD-related healthcare savings from the utilization of cereal fiber for increasing dietary fiber consumption from current levels (**Table 1**) to levels that correspond to the IOM’s adequate intake cut-offs, estimated at 38 g per day for men and 25 g per day for women (Institute of Medicine, 2002). The universal fortification represents the best-case scenario of potential cost savings if all Canadian adults (≥18 years of age) were to consume the adequate quantities of dietary fiber. The optimistic scenario is a medium- to short-term pragmatic estimate of the potential cost savings when 50% of adults increase intakes of dietary fiber. The pessimistic scenario is a practical short- to medium-term estimate of cost savings that could follow the increase in dietary cereal fiber intakes among 15% of adults. The very pessimistic scenario represents the worst-case estimate when up to 5% of adults make the dietary change.*

Potential T2D and CVD cost savings with each 1 g per day increase in dietary cereal fiber intake are presented in **Tables 8** and **9**, respectively. There, given predicted worst-to-best case scenarios, total annual cost savings ranged between CAD$2.6 and $51.1 million for T2D, and $4.6 and $92.1 million for CVD.

**Table 8:** Potential savings in type 2 diabetes cost among Canadian adults for each 1 g per day increase in intakes of dietary cereal fiber (CAD $million).

	Scenario			
Cost category	Universal	Optimistic	Pessimistic	Very pessimistic
Direct cost savings				
Hospital	2.1	1.1	0.3	0.1
Physician	13.0	6.5	2.0	0.7
Drugs	32.1	16.0	4.8	1.6
Total direct cost savings	47.2	23.6	7.1	2.4
Indirect cost savings				
Mortality	0.3	0.2	0.1	0.0
Morbidity	3.6	1.8	0.5	0.2
Total indirect cost savings	3.9	1.9	0.6	0.2

**Total cost savings**	**51.1**	**25.5**	**7.7**	**2.6**

*The universal fortification represents the best-case scenario of potential cost savings in all Canadian adults (≥18 years of age). The optimistic scenario is a medium- to short-term pragmatic estimate of the potential cost savings in 50% of adults. The pessimistic scenario is a practical short- to medium-term estimate of cost savings that could follow the 1 g per day increase in dietary cereal fiber intakes among 15% of adults. The very pessimistic scenario represents the worst-case estimate when up to 5% of adults increase their dietary cereal fiber intakes by 1 g per day.*

**Table 9:** Potential cardiovascular disease cost savings among Canadian adults for each 1 g per day increase in intakes of dietary cereal fiber (CAD $million).

	Scenario			
Cost category	Universal	Optimistic	Pessimistic	Very pessimistic
**Direct cost savings**				
Hospital	9.5	4.8	1.4	0.5
Drugs	50.3	25.1	7.5	2.5
Physician care	27.7	13.8	4.2	1.4
Other direct	0.3	0.1	0.0	0.0
**Total direct cost savings**	**87.8**	**43.9**	**13.2**	**4.4**
**Indirect cost savings**				
Mortality	1.1	0.5	0.2	0.1
Morbidity	3.2	1.6	0.5	0.2
**Total indirect cost savings**	**4.3**	**2.1**	**0.6**	**0.2**

**Total cost savings**	**92.1**	**46.0**	**13.8**	**4.6**

*The universal fortification represents the best-case scenario of potential cost savings in all Canadian adults (≥18 years of age). The optimistic scenario is a medium- to short-term pragmatic estimate of the potential cost savings in 50% of adults. The pessimistic scenario is a practical short- to medium-term estimate of cost savings that could follow the 1 g per day increase in cereal dietary fiber intakes among 15% of adults. The very pessimistic scenario represents the worst-case estimate when up to 5% of adults increase their dietary cereal fiber intakes by 1 g per day.*

Summarized in **Tables 10** and **11** are the predicted economic savings when cereal fiber was used to incrementally increase Canadian adult’s current dietary fiber intakes to 20, 25, 30, and 35 g per day for men and women alike. For T2D, the very pessimistic, pessimistic, optimistic, and universal scenarios’ total savings ranged from CAD$6.8 to $45.2 million, $20.5 to $135.5 million, $68.4 to $451.5 million, and $136.8 to $903.0 million, respectively (**Table 10**). Likewise, savings in CVD healthcare and related costs were estimated to range between CAD$12.3 and $81.4 million (very pessimistic), $37.0 and $244.2 million (pessimistic), $123.3 and $813.9 million (optimistic), and $246.7 million and $1.6 billion (universal) with increasing the current fiber intakes of Canadian adults to 20, 25, 30, and 35 g per day, respectively (**Table 11**).

**Table 10:** Summary of potential total savings in type 2 diabetes costs among Canadian adults with incremental increases in intakes of dietary cereal fiber (CAD $million).

	20 g per day	25 g per day	30 g per day	35 g per day
Universal	136.8	392.2	647.6	903.0
Optimistic	68.4	196.1	323.8	451.5
Pessimistic	20.5	58.8	97.1	135.5
Very pessimistic	6.8	19.6	32.4	45.2

*Based on differences from current intakes of dietary fiber (**Table 1**), the universal fortification represents the best-case scenario of potential cost savings if all Canadian adults (≥18 years of age) were to use cereal fiber to increase daily fiber intakes to 20, 25, 30, or 35 g per day for men and women. The optimistic scenario is a medium- to short-term pragmatic estimate of the potential cost savings when 50% of adults adopt the incremental increases in intakes of dietary fiber. The pessimistic scenario is a practical short- to medium-term estimate of cost savings that could follow the incremental increases in dietary fiber intakes among 15% of adults. The very pessimistic scenario represents the worst-case estimate when up to 5% of adults make the dietary changes.*

**Table 11:** Summary of potential total savings in cardiovascular disease costs among Canadian adults with incremental increases in intakes of dietary cereal fiber (CAD $million).

	20 g per day	25 g per day	30 g per day	35 g per day
Universal	246.7	707.0	1,167.4	1,627.8
Optimistic	123.3	353.5	583.7	813.9
Pessimistic	37.0	106.1	175.1	244.2
Very pessimistic	12.3	35.4	58.4	81.4

*Based on differences from current intakes of dietary fiber (**Table 1**), the universal fortification represents the best-case scenario of potential cost savings if all Canadian adults (≥18 years of age) were to use cereal fiber to increase daily fiber intakes to 20, 25, 30, or 35 g per day for men and women. The optimistic scenario is a medium- to short-term pragmatic estimate of the potential cost savings when 50% of adults adopt the incremental increases in intakes of dietary fiber. The pessimistic scenario is a practical short- to medium-term estimate of cost savings that could follow the incremental increases in dietary fiber intakes among 15% of adults. The very pessimistic scenario represents the worst-case estimate when up to 5% of adults make the dietary changes.*

## Discussion

Using a cost-of-illness analysis, this study assessed the potential savings in costs of T2D and CVD within the Canadian healthcare system following higher intakes of dietary cereal fiber among adults. The potential economic benefits that resulted from improved intake of cereal fiber were significant. Specifically, if between 5 and 100% of the Canadian adults were to utilize cereal fiber to adopt intakes of dietary fiber that correspond to the IOM’s adequate intake levels (Institute of Medicine, 2002), approximately CAD$36 to $720 million and CAD$65 million to $1.3 billion would accrue as total annual savings in healthcare and related costs associated with T2D and CVD, respectively. These savings are substantial relative to the current healthcare budget in Canada.

The rising costs of healthcare are a growing concern, especially in Canada where direct costs of treating disease are borne largely by an increasingly aging public. Any opportunity to reduce these costs should be fully studied. The health benefits of increased dietary fiber intakes are well-recognized. Accumulating evidence suggests protection against a range of major public health concerns, including diabetes (Murtaugh et al., 2003; Schulze et al., 2007), CVD (Liu et al., 1999; Pereira et al., 2004), obesity (Liu et al., 2003), cancers (Aune et al., 2011, 2012), and gastrointestinal disorders (Petruzziello et al., 2006), all of which place substantial burdens on healthcare resources in Canada and, likewise, around the globe. Still, very little knowledge is available on the economic benefits of greater habitual or recommended intakes of fiber. We are unaware of other economic analyses that have previously assessed the potential savings in T2D- and CVD-related costs when dietary fiber consumption is increased. Although, a few recent studies have reported the valuable effects of increased fiber intakes on healthcare costs for functional constipation in the US (Schmier et al., 2014) and Europe (Schmier et al., 2015).

Clinical and epidemiological research continues to delineate the benefits of foods and nutrients with respect to general health as well as reduced risk of chronic disease. Such data are useful for developing and adjusting dietary recommendations. Given that the definition of dietary fiber is broad in scope and can include indigestible carbohydrate from a variety of dietary sources (Health Canada, 2012a), it is reasonable that different fibers from diverse foods impose unique health benefits. Based on supportive data from published risk analyses, this economic valuation focused on cereal fiber as a means of reducing future incidence of T2D and CVD. It is important to note, however, that this analysis does not support a reductionist approach to healthful diets; where cereal fiber is identified as the only fiber that can facilitate health and economic benefits. For example, while cereal fibers are efficacious for decreased T2D and CVD risk, other fibers, and the foods containing them, such as fruits and vegetables, are widely accepted as part of healthy diet. Accordingly, this analysis modeled the addition of cereal fiber to current fiber intakes (19.1 g for men and 15.6 g for women), which recognizes a baseline dietary fiber consumption from various dietary sources. This study does support, however, that health as well as economic benefits could arise if Canadians impose more healthful lifestyles. From this and other nutritional economic data (McCarron and Heaney, 2004; Dall et al., 2009; Bibbins-Domingo et al., 2010; Gyles et al., 2010; Schmier et al., 2014, 2015), it is logical that as healthy lifestyle habits compound, societal benefits will also grow in parallel.

For T2D and CVD, results from this study complement those from Schmier et al. (2014, 2015) and highlight the importance of communicating such health benefits of dietary fiber to the general public and various stakeholders. For the food industry, emphasis on the provision of higher-fiber foods could help facilitate a national economic benefit. Government and healthcare professionals are crucial for implementing strategies that capitalize on potential success of realizing any economic savings from increasing fiber consumption in Canada. Policy makers will need to set clear guidelines that facilitate a high degree of utility for consumers by setting guardrails around the type(s) of health claims permitted that ensure consistent and credible message is received by consumers. Finally, educators and healthcare providers will play a crucial role in emphasizing the importance of dietary fiber to patients and the public.

While this cost-of-illness analysis demonstrated the potential for substantial healthcare savings with increased consumption of cereal fiber, it is reasonable that there would be costs associated with the implementation of initiatives that impose such dietary changes for Canadians. These costs would majorly be incurred by two of the aforementioned stakeholders: government(s) and the consumer. Government spending would manifest as programs that promote the consumption foods that impact the Canadian healthcare economic landscape, such as dietary sources of cereal fiber. Initiatives could include increased emphasis for cereal-derived fiber within current programs such as Canada’s Food Guide (Health Canada, 2011) as well as independent marketing campaigns. As a partnership with the food industry, the latter strategy has been utilized to increase Canadians’ awareness and use of the Nutrient Facts Table on Canadian food labels (Government of Canada, 2015). While new and refreshed programs would be of value for heightening dietary awareness of foods that could impact healthcare costs, governments and regulatory agencies across jurisdictions, including Canada, have already implemented a regulatory framework that promote the consumption of specific cereal fibers that are directly linked with reduced risk factors for CVD. For example, Health Canada, The Food and Drug Administration, The European Commission, and Food Standards Australia New Zealand permit front-of-pack health claims on foods that communicate the presence of defined threshold levels of oat or barley-derived beta-glucan and their ability to lower circulating cholesterol levels (Health Canada, 2010, 2012b; European Commission, 2011, 2012; Food and Drug Administration, 2013; Zealand FSAN, 2014). Perhaps, further funding allocation is required to bring increased awareness to specific health claims in market, as well as increased use of sources of cereal fiber by industry.

For the second stakeholder, the consumer, the most significant costs incurred would be those required for imposing dietary change. While this analysis provided cost savings per g increase in cereal fiber consumption, it is ideal if increased consumption of cereal fiber among Canadians surpassed one g per day. That being said, within Canada and other regions, foods with cereal fiber are ubiquitous within the food supply as foods with whole grain and/or added bran. Moreover, dietary recommendations across jurisdictions promote the consumption of whole grains, which are sources of cereal fiber (United States Department of Agriculture, 2010; Health Canada, 2011; Australian Government, 2013). Whole grains are also primary components of healthful traditional diets such as the Mediterranean and Nordic Diet (Jensen and Poulsen, 2013; Gerber and Hoffman, 2015). For some consumers, there could be a cost to consuming a healthier diet that incorporates efficacious increases in specific nutrients such as dietary cereal fiber (Jensen and Poulsen, 2013). However, when the biological need to consume food is combined with minimal consumption of foods or constituents that counteract the health benefits of cereal fiber, these costs to the consumer would be marginal; and align with the costs realized if the populace fully embraced regional dietary guidance from governments, dietitians, and other healthcare practitioners.

In addition to government and consumers, industry is also a critical stakeholder for facilitating dietary changes among Canadians, including increased consumption of cereal fiber. Given that it is the food industry that manufactures and supplies a significant proportion of the foods that comprise the diets of Canadians, it is reasonable that increased demand for sources of dietary cereal fiber would also incur an upfront cost to industry. These costs would come from new product innovation, renovation of existing products, manufacturing, shipping, listing fees, and marketing. While upfront costs to the food industry are realized, strong demand for nutritious food with cereal fiber will drive continued support for the development of foods that contain whole grains and/or added bran. Governments already impose regulations that outline thresholds regarding the minimum levels of dietary fiber required to make “source of fiber” claims of varying magnitude (European Commission, 2006; Zealand FSAN, 2014; The Canadian Food Inspection Agency, 2015). Meeting these threshold levels is crucial for permitting industry to communicate healthful food attributes to the Consumer. In addition to artisan or unprocessed foods that contain fiber from cereal grains, epidemiological studies that demonstrate an inverse association between cereal fiber and risk of diabetes, CVD, and mortality include foods manufactured by the food industry, such as fiber-containing ready-to-eat cereals (Cho et al., 2013; Huang et al., 2015). Continued emphasis from nutrition leaders in the private and public sectors as well as food innovation that facilitates widespread consumption of foods with efficacious levels of cereal fiber will be critical for widespread healthcare cost savings from dietary change.

As discussed previously, to our knowledge, this work is the first to examine the potential savings in costs to the Canadian healthcare system attributed to higher intakes of dietary fiber manifesting in lower rates of T2D and CVD. We utilized the most recent literature and national databases for findings pertaining to identification of the consumer trends and healthcare costs. Developing three sets of analyses enabled the prediction of healthcare cost savings that are mostly communicable to policy makers, dietitians, and other healthcare providers. For example, while IOM target levels of fiber intakes are likely most beneficial for government policy makers and healthcare associations that set guidelines for clinical practice, incremental targets are mainly beneficial to the general public and healthcare practitioners who focus on a stepwise approach for assisting patients with increasing fiber consumption.

This study also has limitations. Estimates of risk reduction per g cereal fiber were derived from studies that gave prescribed levels of cereal fiber. Thus, it was assumed that the relationship between cereal fiber dose and risk reduction was linear. However, in some cases a linear dose effect may not exist across all levels of cereal fiber consumed; and, in those instances, thresholds for cereal fiber intake must be met for increased risk reduction. Recently, data from the InterAct Consortium demonstrated that an inverse effect of cereal fiber on T2D risk was linear with a steeper slope at >10 g/day (InterAct Consortium, 2015). That being said, the cost-of-illness analysis in this study utilized conservative estimates of risk reduction that were generated from meta-analyses and were assumed to be constant across all dosages of cereal fiber. Moreover, fiber-containing foods that are recommended by healthcare practitioners and dietary guidelines typically contain substantial levels of dietary fiber that would assist consumers in consuming >1 g cereal fiber per serving and per day.

Another limitation of the present work may relate to the fact that isolating the reported health benefits of dietary cereal fiber intakes from other associated lifestyle and environmental factors is difficult. Fiber intake from certain sources, such as fruits and vegetables, usually associates with better socio-economic status (Dubowitz et al., 2008; Boylan et al., 2011), such as higher education and income, thus possibly healthier overall environments. To what extent these other lifestyle factors that associate with higher fiber intakes serve as confounders in our model remains to be assessed.

## Conclusion

Given the economic burden of T2D and CVD in Canada and worldwide, the novel findings of this analysis shed light on the economic valuation of optimal dietary fiber intakes and reach beyond the well-established direct health benefits. Strategies to increase the consumers’ general knowledge of the recommended intakes of dietary fiber, and benefits of cereal fiber, as part of healthy diet and overall lifestyle, and to facilitate stakeholder synergy, are highly warranted. Ultimately, such evidence-based strategies are expected to enable better management of healthcare and related costs associated with T2D and CVD.

## Author Contributions

MA and CG designed the economic model, reviewed the literature, and carried out the monetary analyses. MA interpreted the data and drafted the manuscript with assistance from CM. CM, JC, and PJ conceived the study question. All authors contributed to the interpretation of data, critically reviewed the manuscript for important intellectual content, and approved the final version.

## Conflict of Interest Statement

Funding from Kellogg Canada Inc. directly supported the efforts of Mohammad Abdullah, Collin Gyles, Jared Carlberg, and Peter Jones. The funding organization had no role in defining the study design; in the collection, analysis, or interpretation of data; in the writing of the manuscript; or in the decision to submit the manuscript for publication. At the time of the manuscript’s inception and acceptance for publication, CM was an employee of Kellogg Canada. The authors declare that they have no other competing interests.

## Abbreviations

CIHI: Canadian Institute for Health Information
CVD: cardiovascular disease
EBIC: Economic Burden of Illness in Canada
IOM: Institute of Medicine
NHEX: National Health Expenditure Trends
T2D: type 2 diabetes.
